# Enhanced Heat Dissipation Performance of Automotive LED Lamps Using Graphene Coatings

**DOI:** 10.3390/polym14010050

**Published:** 2021-12-23

**Authors:** Tun-Ping Teng, Wei-Jen Chen, Chun-Hsin Chang

**Affiliations:** Undergraduate Program of Vehicle and Energy Engineering, National Taiwan Normal University, No. 162, Sec. 1, He-ping E. Road, Da-an District, Taipei City 10610, Taiwan; tube5711@ntnu.edu.tw (T.-P.T.); kylechen@ntnu.edu.tw (W.-J.C.)

**Keywords:** automotive LED lamps, combined junction, graphene heat-dissipating coatings (GNHC), illuminance efficiency, thermal resistance

## Abstract

Graphene heat-dissipating coating (GNHC) of 0.6 wt % GN concentration is utilized to promote the cooling performance of automotive light-emitting diode (LED) lamps. Three cases are studied as follows: Case 0 is the original automotive LED lamp as the baseline. Case 1 is to apply GNHC to reduce the thermal resistance of the junction surfaces between the components of automotive LED lamps. The aluminum fin radiator of Case 1 is further coated with GNHC on the surface that becomes Case 2. The spectrum, illuminance, power consumption, and surface temperature are measured at different ambient temperatures (*T_a_*) to fully evaluate the feasibility of applying GNHC to improve cooling performance and the impacts on the related characteristics of automotive LED lamps. The results show that the maximum illuminance efficacy of Case 1 and Case 2 with high beam, irradiation angle of 0 degrees, and *T_a_* of 80 °C is 11.03% and 8.70% higher than that of Case 0, respectively. The minimum temperature difference of heat dissipation path of Case 1 and Case 2 with high beam, irradiation angle of 90 degrees, and *T_a_* of 80 °C is 6.41% and 5.33% lower than that of Case 0, respectively, indicating GNHC as a promising coating material for improving the cooling performance of automotive LED lamps.

## 1. Introduction

With the improvement of automotive lighting technology and increasing demand, light-emitting diode (LED) lamps have gradually become the mainstream configuration for automotive headlight lighting in recent years. Using LED lamps has several advantages—including low power consumption, low waste heat generation, long service life, high lighting intensity, and high luminous efficiency—while complying with relevant laws and regulations for vehicles [1,2,3]. However, the maximum junction temperature (*T_j_*) of automotive LED chips is usually around 150 °C [4]. When the *T_j_* exceeds such a limit, the luminous performance and life of the LED chips will be seriously affected [4,5,6]. In addition, the ambient temperature (*T_a_*) will affect the cooling performance of the LED lamps and thus the *T_j_* of the LED chips. LED headlights have the highest brightness requirements, thus resulting in the highest power consumption and heat generation among all automotive LED lamps. Therefore, they are the priority in terms of automotive lamp thermal management. Studies have shown that the ambient temperature within the engine room of an internal combustion engine vehicle can reach up to 80 °C while having an outside air temperature of 35 °C. This high inside *T_a_* will also harm the use of LED lamps [7,8].

Many researchers have proposed effective solutions to address the cooling problem of automotive LED lamps, including forced-circulation air-cooled radiators [9,10], high-efficiency heat dissipation fins [5], radiators with special geometry and structure [11], heat pipe radiators [10,12], etc. All of these are proved to reduce *T_j_* and (or) the thermal resistance of the heat dissipation path of automotive LED lamps.

Since an LED lamp is composed of multiple components, the generated waste heat from the heat source must be transferred through the path to the radiator as the heat sink before being dissipated ambiently. As a result, the tightness of the junction surfaces between components will greatly affect the overall heat transfer coefficient. Therefore, the thermal interface materials (TIMs) with high thermal conductivity (*k*) must be used on these junction surfaces to improve the heat dissipation performance of the electronic components [13,14,15]. To achieve this goal, adding high-*k* fillers to traditional TIMs is one of the important methods to enhance the *k* of TIMs. Among all of the possible fillers, ceramics [16,17,18,19] and metals [19,20,21] are commonly selected materials. Typically, ceramic fillers have good electrical insulation properties but low *k*, while metal fillers have high *k* but poor electrical insulation and are easily oxidized.

In recent years, carbon-based nanomaterials, such as carbon nanotubes (CNTs) and graphene (GN)-based materials are often used as fillers for TIMs because of their extremely high *k* and special morphology [22,23,24,25,26,27]. The substrate of these thermal interface materials includes cellulose [22], natural rubber [23], non-curing mineral oil matrix [24], polydimethylsiloxane (PDMS) [25,27], and epoxy resin [26]. Some researchers mixed CNTs and GN- based materials with other materials as the filler of TIM and discovered a more than 10-times increase on *k* due to their synergistic effect [28,29,30,31]. Therefore, CNTs and GN- based materials have great potential as fillers for TIMs. However, most carbon-based nanomaterials are conductive, making the filling ratio an important parameter that must be controlled to maintain a good electrical insulation property.

In the past, most LED lamp cooling research focused on the effect of heat dissipation methods on the optical characteristics and the cooling of the components. However, the relationship between improving the heat dissipation capacity and power consumption of automotive LED lamps is rarely mentioned. This study utilizes GN and epoxy resin as fillers and substrates of the graphene heat-dissipating coatings (GNHC), respectively. The GNHC is then applied on the component junction surfaces and exterior of the aluminum fin radiator of an LED lamp of headlights to verify its performance as TIM and as a heat dissipation enhancement coating. The spectrum, illuminance, power consumption, and surface temperature of each point of the automotive LED lamp under different *T_a_* are measured to evaluate the feasibility of applying GNHC comprehensively.

## 2. Preparation of GNHC

In this study, a single-component epoxy resin (ETERKYD NP1023-R-50-1, Eternal Materials Co. Ltd., Taoyuan City, Taiwan) [32] was used as the substrate material for the GNHC. This epoxy resin can be hardened with an 80 °C, 30 min duration heating process. The epoxy resin’s thermogravimetric analysis (TGA) showed that the thermal decomposition temperature of the hardened epoxy resin could exceed 300 °C, which is significantly higher than the limit temperature of 175 °C for automotive LED chips [4]. The commercially available GN (FNG-320-MF-OL505, G. I. Business Co. Ltd., New Taipei City, Taiwan) [33] added to the GNHC is prepared by the mechanical exfoliation method, and the lipophilic few-layer GN is formed after surface modification treatment. According to the supplier’s specifications, the thickness, diameter, and purity of the GN sheet are less than 3 nm, 0.1–20 μm, and greater than 98%, respectively.

**Figure 1:**
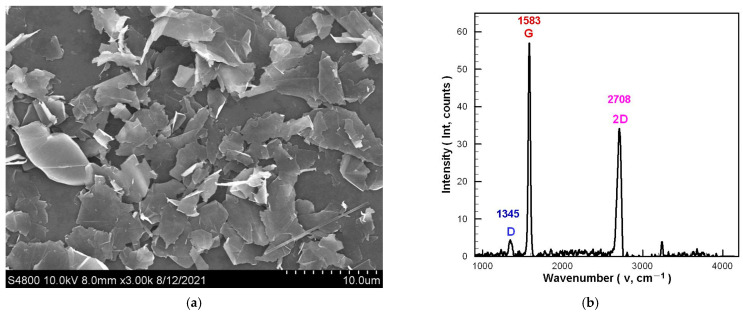
Apparent morphology and material analysis of GN: (**a**) SEM image; (**b**) Raman spectra.

Figure 1 is the apparent morphology and material analysis of the GN. Figure 1a shows a scanning electron microscope (SEM; S4800, Hitachi, Saitama, Japan) image of the GN. Its apparent morphology is flaky, and the sheet diameter is within the specifications declared by the supplier. Figure 1b is the Raman spectra of the GN tested by the Raman spectroscopy (532.15 nm, NRS4100, Jasco, Tokyo, Japan). Like a typical GN, the G peak and 2D peak appear around 1582 cm^−1^ (1583 cm^−1^) and 2700 cm^−1^ (2708 cm^−1^), respectively. Furthermore, the *I_2D_/I_G_* of single-layer GN is greater than 1, and the *I_2D_/I_G_* of few-layer GN is greater than 0.5 [34,35,36]. Since the GN used in this study has *I_2D_/I_G_* = 0.599, it can be confirmed that the few-layer GN meets the specifications declared by the supplier because GN exhibits a flake-powder morphology with a defect peak (D peak) at 1345 cm^−1^, and its *I_D_/I_G_* = 0.077.

**Figure 2:**
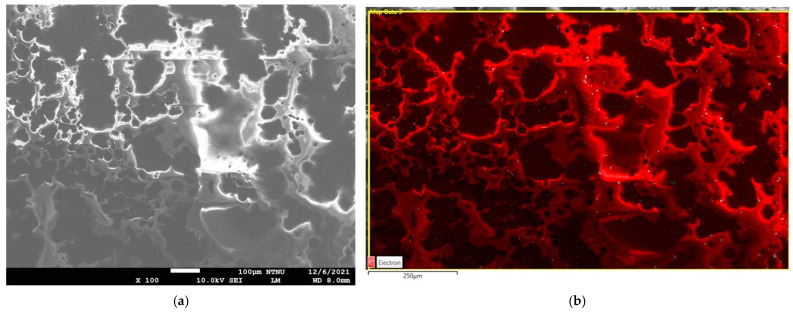
GNHC coating analysis: (**a**) SEM image; (**b**) elemental mapping of EDS.

In the beginning, the GN is weighed and soaked in isopropanol (IPA, purity: 99.5%; Union Chemical Works Ltd., Hsinchu, Taiwan) to prepare a 2.0 wt % GN-IPA suspension. The 2.0 wt % GN-IPA was evenly dispersed through multiple process equipment, including electromagnetic stirrer (PC420D, Corning, NY, USA), ultrasonic bath (5510R-DTH, Branson, St. Louis, MO, USA), and high-speed homogenizer (YOM300D, YOTEC, Hsinchu, Taiwan). After that, the GN-IPA is poured into the epoxy resin at a weight ratio of 30% and evenly mixed with a combined usage of hand stirring, electromagnetic stirrer, and an ultrasonic bath. As a final product, the concentration of GN in the GNHC is 0.6 wt %. Theoretically, a higher GN concentration results in increased thermal conductivity and viscosity of GNHC, which the latter hinders coating operation. Therefore, we used the trial-and-error method (brush coating) to test the upper limit of the suitable GN concentration. Finally, the upper limit of the GN concentration in GNHC that can be smoothly coated on the aluminum substrate is 0.6 wt %. Figure 2a,b are respectively the SEM (JSM-7610, Jeol, Tokyo, Japan) image and elemental mapping of energy-dispersive X-ray spectroscopy (EDS) for the GNHC coating on an aluminum substrate. It can be observed from the SEM image that the GN is connected in a net shape and partially agglomerated. However, it is difficult to avoid the agglomeration problem completely because GN with flaky morphology is dispersed in the epoxy resin with higher viscosity. In addition, it can be seen from the elemental mapping of EDS that the GN is roughly evenly distributed in the epoxy resin, indicating that the dispersion technology used in this study is acceptable.

## 3. Experimental Design and Implementation

### 3.1. Description of the Experimental Subject

**Figure 3:**
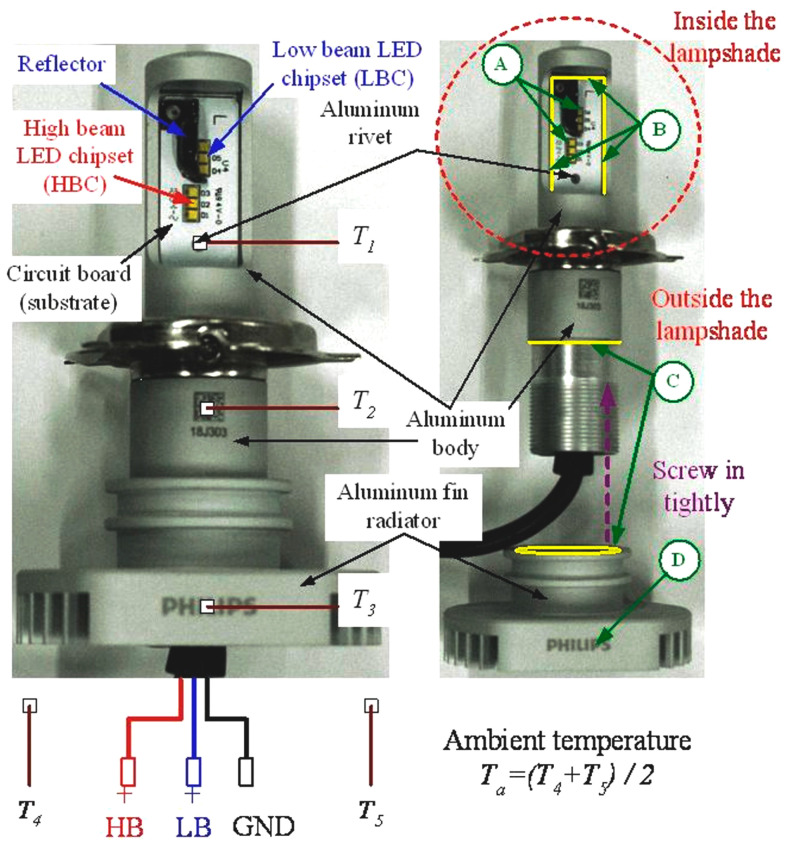
Photograph of the automotive LED lamp.

Figure 3 shows the automotive LED lamp (6000 K pure white LUXEON; LED-HL [≈H4], PHILIPS, Shanghai, China) used for testing in this study. This automotive LED lamp is utilized as the headlight of the car. The rated supply voltage, input current, and power consumption for this automotive LED lamp are 13.2 V, 1.2 A, and 15.5 W, respectively. On both sides of the lamp, there exists one LED module consisting of one low beam LED chipset (LBC) and one high beam LED chipset (HBC), where each LED chipset consists of three LEDs. There is a reflector under the LBC on both sides to control the irradiation direction of the LBC. The LED module on both sides is riveted on the aluminum substrate. All thermocouples (1/0.2 mm × 2C, T-type, accuracy: ±0.75%) used are of the same form and have been calibrated before the experiment. Three thermocouples are installed on the substrate (circuit board, *T*_1_), aluminum body (*T*_2_), and aluminum fin radiator (*T*_3_), respectively, to measure the temperature at these points. The thermocouple that measures the temperature of the substrate was installed on the aluminum rivets. In addition, two thermocouples (*T*_4_ and *T*_5_) were installed in the test space of the automotive LED lamp to measure the *T_a_* of the experiment. The average value of the measurement data of the two thermocouples was defined as the averaged *T_a_*.

### 3.2. Thermal Resistance Analysis and Experimental Case Setting

**Figure 4:**
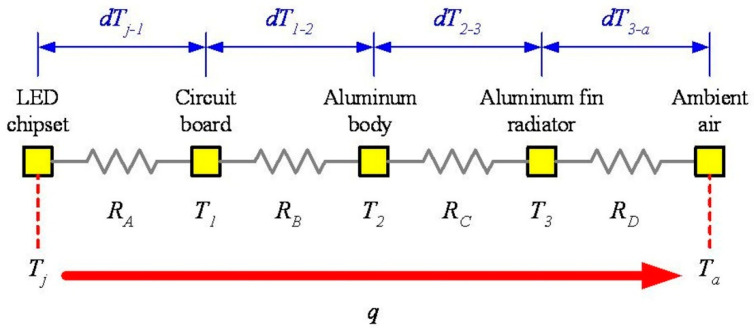
Schematic diagram of the thermal resistance of the LED lamp.

As shown in Figure 3, most of the heat generated by the LED chips will be transferred to the rear aluminum fin radiator before being dissipated to the ambient air since a lampshade will cover the LED chips in normal operation. The schematic diagram illustrating the equivalent thermal resistance of the heat dissipation path of the automotive LED lamp is shown in Figure 4. It is worth mentioning that the model neglects the thermal resistance of components itself since they are all metallic materials except the LED chipset. The calculation of the total thermal resistance (*R_T_*) and the thermal contact resistance (*R*) between individual components of the automotive LED lamp is shown in Equations (1) and (2). The temperature difference (*dT*) is proportional to the *R* under the same heat flux (*q*). The *q* is the product of the conversion coefficient (*e*: power consumption into thermal power) and the power consumption (*P*). The entire automotive LED lamp is composed of multiple components. Since the tightness of the junction gap and surface strongly affects the thermal contact resistance. Therefore, applying the GNHC as the TIMs on these junction gaps and surfaces should reduce the thermal contact resistance and improve the heat dissipation capacity of the automotive LED lamp.

On the other hand, since GN has high emissivity, coating the GNHC on the aluminum fin radiator should improve the aluminum fin radiator’s radiation performance. Furthermore, coating GNHC on the aluminum fin radiator can change the surface morphology and roughness of the aluminum fin radiator, which may help convective heat transfer performance due to the increased surface area. Furthermore, the GNHC prepared in this study has proper fluidity and high-temperature tolerance. It can be directly applied to the assembly gap by brush method then dripped to the junction to be coated without disassembling the components. Therefore, GNHC can be used as both TIMs and heat dissipation coatings simultaneously. As a result, three experiments are conducted as follows to test the hypothesis mentioned above:

1. Case 0: The original automotive LED lamp without any GNHC (baseline).
2. Case 1: Applying the GNHC on the junction gaps and surfaces of the substrate to the aluminum body and the aluminum body to the aluminum fin radiator (refer to the B and C position with yellow marks in Figure 3) by brush method. An 80 °C, 60 min duration heating process is performed for GNHC hardening.
3. Case 2: Same as Case 1, plus a GNHC coating applied on the surface of the aluminum fin radiator (refer to D position in Figure 3) by brush method. An identical hardening procedure is performed as in Case 1.
  (1) $$R_{T}=\frac{\left(T_{j}-T_{a}\right)}{q}=\frac{dT_{j-a}}{q}=\frac{dT_{j-1}}{q}+\frac{dT_{1-2}}{q}+\frac{dT_{2-3}}{q}+\frac{dT_{3-a}}{q}=R_{A}+R_{B}+R_{C}+R_{D}$$
  (2) q=e×P

### 3.3. Experimental Procedure

**Figure 5:**
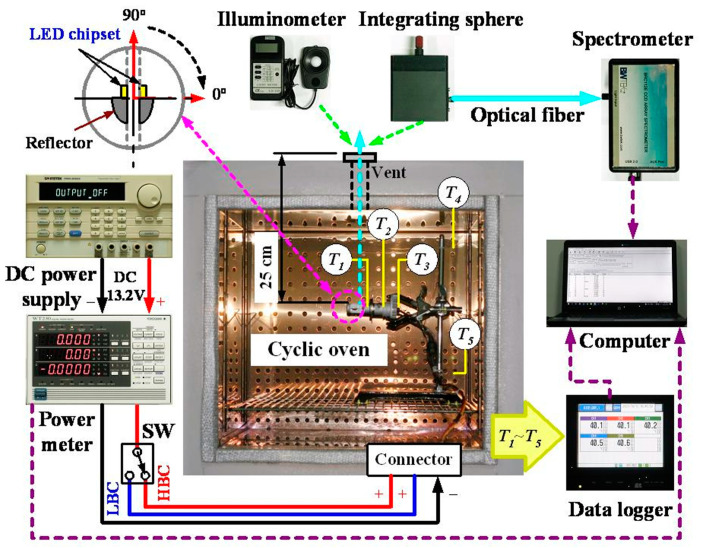
Schematic diagram of the experimental measurement system.

The illuminance, spectrum, and power consumption at different *T_a_* (40, 60, and 80 °C) are measured to evaluate the impact of applying the GNHC under different scenarios (Case 0 to Case 2). The automotive LED lamp is fixed on the holder and placed in a temperature-controllable cyclic oven (internal dimensions: W60 × D50 × H50; DH-600N, YOTEC, Hsinchu, Taiwan) to test the above-mentioned experimental parameters. Figure 5 is a schematic diagram of the experimental measurement system. The center of the LED matrix is aligned with the air vent located at the top of the oven for illuminance and spectrum measurement purposes. The measurement distance is about 250 mm. The illuminance and spectrum (350–800 nm) of the automotive LED lamp is measured by an illuminance meter (LX-103, Lutron, Taipei City, Taiwan; accuracy: ±5.0%) and a spectrometer (wavelength range: 200–850 nm; BRC112E-V Quest^TM^X, B&W Tek, Plainsboro, NJ, USA) with an integrating sphere (ø = 5 cm), respectively. Because the measurement deviation of the illuminance meter is relatively high, each experimental parameter is measured six times and averaged as the final experimental data. The illuminance and spectrum of the automotive LED lamp are measured in two directions by rotating a pre-defined angle (0 and 90 degrees) to understand the influence of different directions and *T_a_*. The components of the automotive LED lamp in the 0 degrees and 180 degrees directions are exactly the same, and the positions are symmetrical. Therefore, only one of them is selected for testing.

A digital programmable power supply (0–30 V/6 A; PSM-6003, GW Instek, New Taipei City, Taiwan; accuracy: ±0.05% + 10 mV/0.2% + 10 mA) with a constant voltage of 13.2 V is selected as the power supply of the LED lamp. It is equipped with a power analyzer (WT230, YOKOGAWA, Tokyo, Japan; accuracy: ±0.2%) to continuously record related power data with a sampling time of 5 s. A data logger (TRM20, TOHO, Kanagawa, Japan; accuracy: ±0.1% + 1 digit) is utilized to record related temperature data continuously with a sampling time of 5 s, too. The power supply of the high beam and the low beam is controlled by a switch (SW). Each experimental parameter is conducted for 20 min after the *T_a_* is stabilized at the set-point. The temperature and power consumption of the automotive LED lamp are the averages of the last three minutes of the measurement data as the steady-state experimental data (average data from 17th to 20th minutes). The measurement of spectrum and illuminance is completed between the 17th and 20th minutes of the experiment time.

### 3.4. Data Analysis and Relative Uncertainty

To better demonstrate the impacts of applying the GNHC, the relevant experimental data will be compared to Case 0 (original automotive LED lamp without any GNHC) as the baseline. As shown in Equation (3), the experimental data of other Cases (Case 1 and Case 2) and the experimental data (*DT*) of the Case 0 at the same *T_a_* are converted into percentage differences (*DR*) to present the influence of GNHC.

(3) $$DR=\left[(DT_{Case\;1\;or\;2}-DT_{Case\;0})/DT_{Case\;0}\right]\times 100\%$$

The relative uncertainty analysis performed herein involved the calculation of measurement deviations (*d*) of the instrument. According to Equation (4) for standard uncertainty analysis, the range of relative uncertainty of *T*, *dT*, *P*, and illuminance efficacy (*η_lx_*) were ±0.76%, ±1.07%, ±0.29%, and ±5.01%, respectively.

(4) $$u_{d}=\sqrt{{\left(d_{1}\right)}^{2}+{\left(d_{2}\right)}^{2}+\ldots \ldots \ldots +{\left(d_{n}\right)}^{2}}\times 100\%$$

## 4. Results and Discussion

**Figure 6:**
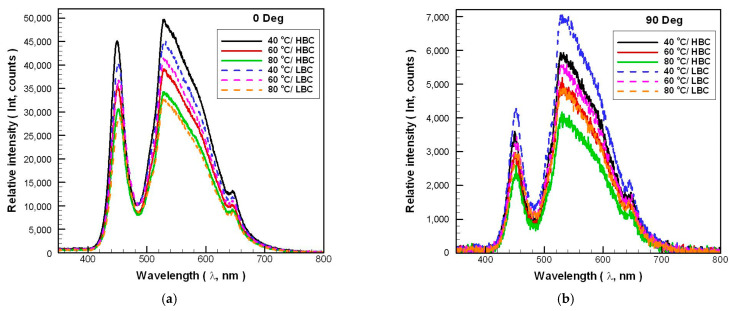
The spectra of Case 0 under different experimental parameters: irradiation angle (**a**) 0 degrees; (**b**) 90 degrees.

Figure 6 shows the test results of the spectra of Case 0 under different experimental conditions. The integration time of spectrometer used to measure the spectrum of the automotive LED lamps with different experimental parameters is 200 ms. Therefore, the relative intensity of the LED’s spectrum distribution is comparable. It can be seen from Figure 6 that regardless of the irradiation angle of 0 degrees or 90 degrees, the relationship between the *T_a_* and the relative intensity of the spectrum is inversely proportional. However, the *T_a_* has almost no effect on the spectrum distribution, and its color temperature meets the manufacturer’s declared specification of 6000 K (the difference in chromaticity coordinates is within 2%) under various *T_a_*. Therefore, the influence of *T_a_* on the spectral characteristics of automotive LED lamps will not be discussed in subsequent cases.

Table 1 lists power consumption (*P*, W) and illuminance (*IL*, lx) for the automotive LED lamps under different experimental conditions. It is shown that the *P* of automotive LED lamps coated with GNHC (Case 1 and Case 2) under the same *T_a_* is slightly higher than Case 0. This phenomenon should be caused by the better heat dissipation of the GNHC-coated automotive LED lamps, which lowers the temperature and equivalent internal resistance of the LED chips, resulting in a higher input current and power under a constant voltage power supply. Furthermore, the *IL* of automotive LED lamps coated with GNHC is also higher than Case 0 under the same *T_a_*, which should be due to higher *P*.

**Figure 7:**
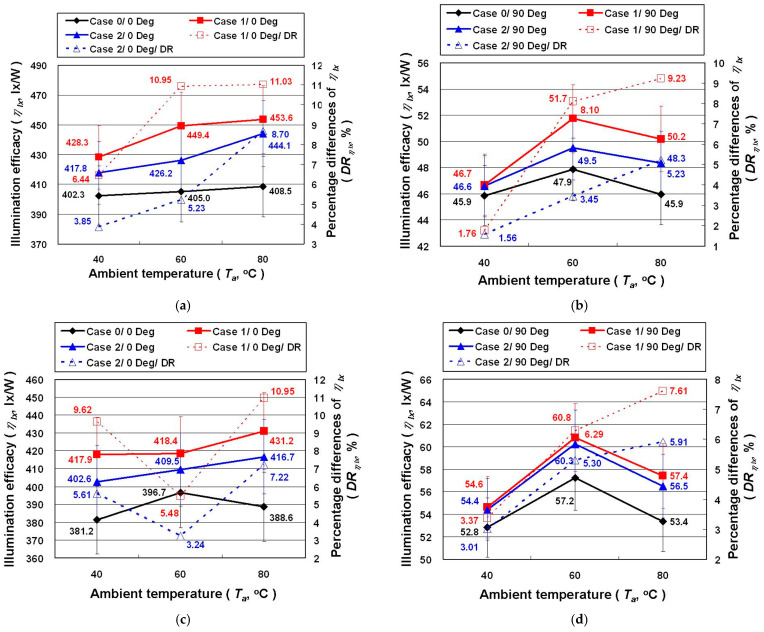
The *η_lx_* and the *DR_ηlx_* of the LED lamps under different experimental parameters: (**a**) high beam/0 degrees; (**b**) high beam/90 degrees; (**c**) low beam/0 degrees; (**d**) low beam/90 degrees.

In order to more clearly show the difference between the *P* and the *IL* of Case 0, Case 1, and Case 2, this study defines the *IL* provided by the unit *P* as the illuminance efficacy (*η_lx_*, lx/W)). Figure 7 shows the *η_lx_* and the percentage differences of *η_lx_* (*DR_η__lx_*, %) for the automotive LED lamps under different experimental conditions. Figure 7 shows that *η_lx_* of the automotive LED lamp is certainly improved in Case 1 and Case 2, where the former is slightly better than the latter. The maximum *η_lx_* of Case 1 and Case 2 with the high beam, irradiation angle of 0 degrees, and *T_a_* of 80 °C is 11.03% and 8.70% higher than Case 0, respectively.

Table 2 lists component temperature (*T*, °C) for the automotive LED lamps under different experimental conditions. It is shown that the substrate temperature (*T*_1_) of the LED lamp coated with GNHC under the same *T_a_* is slightly lower than Case 0. However, the temperature of the aluminum body (*T*_2_) and the aluminum fin radiator (*T*_3_) of the three cases do not have an aligned trend of difference. As shown in Table 1, the *P* of Case 1 and Case 2 is greater than that of Case 0 under the same experimental conditions. Thus, the heat flux of Case 1 and Case 2 supposes to be higher than that of Case 0. Therefore, it is not easy to judge the influence of GNHC on heat dissipation performance of the automotive LED lamps at a single point of component temperature. To further address this topic, the temperature difference between the components, including the difference between the substrate and aluminum body (*dT_1-2_*, °C), the aluminum body and aluminum fin radiator (*dT_2-3_*, °C), and the aluminum fin radiator and ambient air temperature (*dT_3-a_*, °C), are summarized in Table 2 as well. Similarly to what is done in Table 1, percentage differences are calculated as *DR_dT__1-2_*, *DR_dT__2-3_*, *DR_dT__3-a_*, respectively.

As shown in Table 2, the results of *dT_1-2_* and *dT_2-3_* are lower than that of Case 0, confirming the GNHC does help in reducing thermal resistance as TIM. In contrast, the temperature difference between the aluminum fin radiator and the ambient temperature (*dT_3-a_*, °C) is lower in Case 1 than in Case 2, indicating a less satisfactory result. In addition, by comparing the variation of *DR_P_* and *dT_3-a_*, it can be concluded that the GNHC coating applied on the aluminum fin radiator does not improve its heat transfer rate with the ambient air.

**Figure 8:**
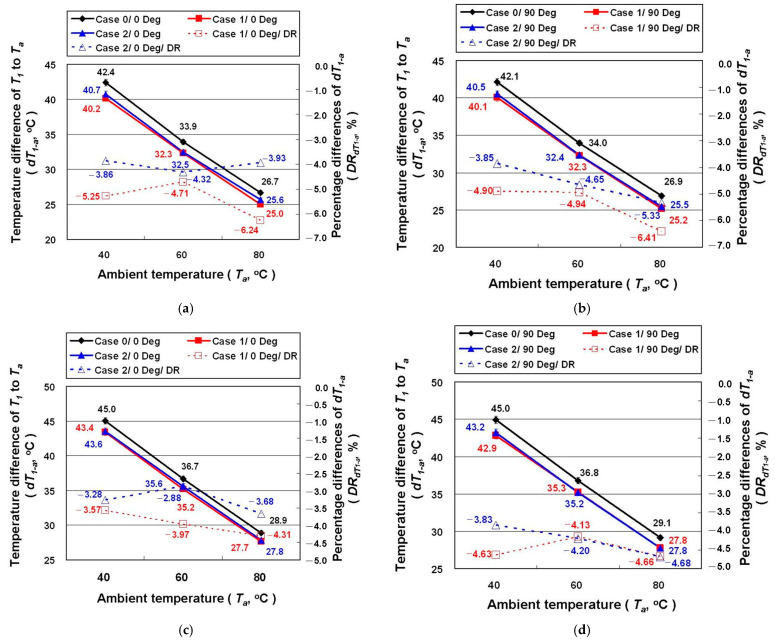
The *dT_1-a_* and the *DR_dT1-a_* of the LED lamps under different experimental parameters: (**a**) high beam/0 degrees; (**b**) high beam/90 degrees; (**c**) low beam/0 degrees; (**d**) low beam/90 degrees.

In order to better illustrate the impact of GNHC coating on the automotive LED lamp in terms of total thermal resistance (*R_T_*), the temperature difference (*dT_1-a_*, °C), and the temperature difference percentage of the heat dissipation path (*DR_dT__1-a_*, %) is plotted as Figure 8. It can be found from Table 1 that the *P* of Case 1 and Case 2 is higher than Case 0 under the same experimental conditions. Thus, a higher *q* can be expected in Case 1 and Case 2 than in Case 0. However, as shown in Figure 8, the *dT_1-a_* of Case 1 and Case 2 is actually lower than that of Case 0, indicating a lower *R_T_*. The relevant results confirm that coating GNHC on the automotive LED lamps can certainly reduce the *R_T_* and improve the cooling performance of the automotive LED lamp. It has to be noticed that the results of Case 1 are better than Case 2. The minimum *dT_1-a_* of Case 1 and Case 2 with the high beam, irradiation angle of 90 degrees, and *T_a_* of 80 °C is 6.41% and 5.33% lower than Case 0, respectively. On the other hand, on average, the lamp power increases by 1.26% and 1.17% in Case 1 and Case 2 compared to Case 0, respectively. Meanwhile, the temperature difference between the aluminum fin radiator and ambient air on average increases by 3.25% and 6.35%, respectively. These results again confirm that there is no benefit in applying the GNHC coating on the radiator.

From the above experimental results and analysis, it can be confirmed that Case 1, in which the LED lamp is coated with the GNHC as the TIMs on the B and C position, results in reduced thermal contact resistance of the automotive LED lamp that leads to the highest illuminance efficiency and cooling performance. However, the increased *q* caused by the increase in the *P* of the automotive LED lamp results in an increased temperature difference between the aluminum fin radiator and the ambient air.

**Figure 9:**
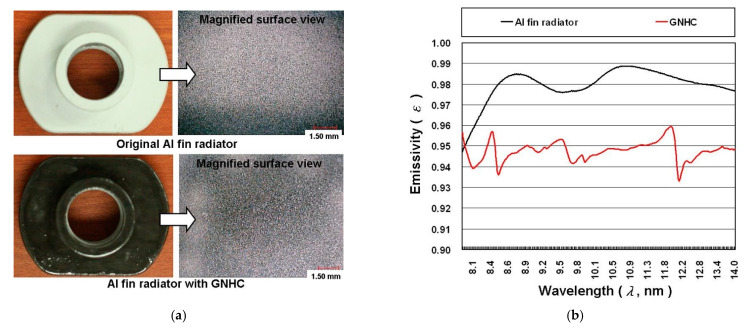
The photographs and *ε* of aluminum fin radiator with and without GNHC: (**a**) actual photographs; (**b**) *ε* curves.

In Case 2, where the aluminum fin radiator is further coated with the GNHC, no improvement can be found in reducing the temperature difference of the heat dissipation path, implying that the additional coating does not reduce the overall thermal resistance. In order to find the causes of this phenomenon that are against our hypothesis before the experiment, the surface of the aluminum fin radiator is examined under a scanning electron microscope. Figure 9 shows the photographs and emissivity (*ε*) of the aluminum fin radiator with and without GNHC. From the magnified image of the surface in Figure 9a, it can be seen that the surface of the GNHC-coated aluminum fin radiator is slightly rougher than that of the original aluminum fin radiator. In addition, the surface of the GNHC coating and the original aluminum fin radiator is measured using a surface profiler (EZSTEP, Force Precision Instrument, New Taipei City, Taiwan) for roughness measurement in four positions. The average surface roughness of the GNHC coating and the original aluminum fin radiator is 1.078 μm and 0.018 μm, respectively. The measurement results confirm that the average surface roughness of the GNHC coating was much higher than that of the original aluminum fin radiator. Although the rougher surface structure might theoretically improve heat convection due to increased surface area; however, the uniformity, morphology, and geometry of the surface roughness will also affect the convective heat transfer performance.

A film thickness meter (MiniTest 700, ElektroPhysik, Köln, Germany) is utilized to measure the ten-points average film thickness of the GNHC coating on the flat surface of the aluminum fin radiator. The average film thickness and relative standard deviations (RSD, %) are 65.3 μm and 23.7%, respectively. An improvement in thickness attenuation and uniformity in the future should further enhance the heat-dissipating performance since the material’s surface morphology distribution and its affinity between the air also affect the convective heat transfer.

However, the relationship of the material surface and the interface properties between radiator and air cannot be verified at present. Furthermore, the concentration of GN contained in the GNHC used in this study is very low, so the *k* of the coating formed by GNHC may be lower than that of the aluminum fin radiator itself, which limits the increase in *k* of the heat conduction path.

Lastly, we use the Fourier-transform infrared spectroscopy (FTIR; IRSpirit, Shimadzu, Kyoto, Japan) to measure the *ε* of 8–14 μm on the surface of the original aluminum fin radiator and the GNHC coating. Figure 8b shows the test results of *ε*. The average *ε* of the original aluminum fin radiator and the GNHC coating surface are 0.98 and 0.95, respectively. Usually, the *ε* of aluminum metal is low, so we believe that the surface of this aluminum fin radiator has been specially treated or coated with a special material to achieve such a high *ε*. As a result, the aluminum fin radiator’s surface radiant heat transfer capacity is actually lowered by the GNHC coating. In addition, the heat-transfer-related factors cause the heat dissipation capacity of the aluminum fin radiator coated with GNHC to be lower than that of the original aluminum fin radiator.

In summary, the above results and analysis show that both Case 1 and Case 2 can improve the heat dissipation performance of automotive LED lamps. However, Case 1 is better than Case 2 in terms of heat dissipation performance. Other topics—such as increasing the GN concentration in GNHC, choosing lower viscosity epoxy resins, thinning the GNHC coatings, and improving coating technique—might be worth further investigation. In addition, the durability of the GNHC is also of interest in future work.

## 5. Conclusions

In this study, a custom GNHC is utilized as TIMs for the junction gaps and surfaces of each component and the heat dissipation coating on the surface of the aluminum fin radiator, attempting to improve the cooling performance and illumination efficiency of the automotive LED lamps. The key findings are summarized as follows:

1. Coating GNHC on the LED lamps can certainly reduce the *R_T_* and improve the heat dissipation performance of the automotive LED lamp.
2. The GNHC performs better as a TIM than as a surface coating for the aluminum fin radiator of the studied automotive LED lamps.
3. The illuminance and power consumption of automotive LED lamps are negatively correlated with the ambient temperature.
4. The maximum *η_lx_* of Case 1 and Case 2 with the high beam, irradiation angle of 0 degrees, and *T_a_* at 80 °C, are 11.03% and 8.70% higher than Case 0, respectively.
5. On average, the lamp power increases by 1.26% and 1.17% in Case 1 and Case 2 compared to Case 0, respectively. Meanwhile, the temperature difference between the aluminum fin radiator and ambient air on average increases by 3.25% and 6.35%, respectively, indicating that applying the GNHC on the radiator’s surface does not help reduce the thermal resistance.
6. The minimum *dT_1-a_* of Case 1 and Case 2 with the high beam, irradiation angle of 90 degrees, and *T_a_* at 80 °C, are 6.41% and 5.33% lower than Case 0, respectively.
7. Increasing the GN concentration in GNHC, choosing lower viscosity epoxy resins, thinning the GNHC coatings, and improving coating technique are potential topics worth further investigation.

## Figures and Tables

**Table 1:** Experimental results of *P* and *IL* of automotive LED lamps.

Irradiation Angle (Deg)	*T_a_* (°C)	Case No.	High Beam				Low Beam			
			*P* (W)	*DR_P_* (%)	*IL* (lx)	*DR_IL_*(%)	*P* (W)	*DR_P_*(%)	*IL* (lx)	*DR_IL_*(%)
0	40	Case 0	12.97	/	5217	/	12.50	/	4765	/
		Case 1	13.16	1.51	5637	8.05	12.60	0.76	5263	10.46
		Case 2	13.29	2.53	5555	6.49	12.58	0.68	5067	6.33
	60	Case 0	11.12	/	4503	/	10.65	/	4225	/
		Case 1	11.23	1.04	5048	12.10	10.81	1.54	4525	7.10
		Case 2	11.31	1.71	4820	7.03	10.83	1.68	4435	4.97
	80	Case 0	9.24	/	3777	/	8.93	/	3472	/
		Case 1	9.41	1.79	4268	13.02	9.06	1.42	3907	12.53
		Case 2	9.37	1.37	4162	10.19	9.06	1.37	3773	8.69
90	40	Case 0	13.21	/	606	/	12.69	/	670	/
		Case 1	13.38	1.29	625	3.08	12.82	1.04	700	4.45
		Case 2	13.28	0.52	619	2.09	12.71	0.19	692	3.21
	60	Case 0	11.21	/	536	/	10.82	/	619	/
		Case 1	11.33	1.07	586	9.26	10.92	0.92	664	7.27
		Case 2	11.33	1.11	561	4.60	10.87	0.49	655	5.82
	80	Case 0	9.29	/	427	/	8.97	/	478	/
		Case 1	9.42	1.38	473	10.73	9.09	1.41	522	9.13
		Case 2	9.42	1.34	455	6.64	9.06	1.06	512	7.04

**Table 2:** Experimental results of temperature and temperature difference of automotive LED lamps: (**a**) high beam; (**b**) low beam.

Irradiation Angle (Deg)	*T_a_* (°C) (Set)	Case No.	*T_1_* (°C)	*T_2_* (°C)	*T_3_* (°C)	*T_a_* (°C)	*dT_1-2_* (°C)	*dT_2-3_* (°C)	*dT_3-a_* (°C)	*DR_dT__1-2_* (%)	*DR_dT__2-3_* (%)	*DR_dT__3-a_* (%)
(**a**)												
0	40	Case 0	82.8	71.2	65.3	40.5	11.6	5.9	24.8	/	/	/
		Case 1	80.5	70.9	65.7	40.3	9.6	5.1	25.4	−17.30	−13.53	2.35
		Case 2	81.0	71.4	66.7	40.3	9.6	4.7	26.4	−16.92	−20.49	6.21
	60	Case 0	94.0	84.4	79.6	60.1	9.5	4.9	19.5	/	/	/
		Case 1	92.6	84.5	80.3	60.3	8.1	4.2	20.0	−14.87	−14.50	2.72
		Case 2	92.7	84.5	80.8	60.3	8.2	3.8	20.5	−14.34	−22.36	5.11
	80	Case 0	106.1	98.4	94.5	79.4	7.7	3.9	15.0	/	/	/
		Case 1	105.0	98.3	95.0	80.0	6.7	3.3	15.0	−13.13	−16.25	−0.10
		Case 2	105.2	98.5	95.5	79.6	6.8	3.0	15.9	−12.36	−23.22	5.42
90	40	Case 0	82.5	71.0	64.2	40.3	11.5	6.7	23.9	/	/	/
		Case 1	80.5	70.5	65.4	40.4	9.9	5.2	25.0	−13.61	−23.22	4.44
		Case 2	81.1	71.1	66.5	40.5	10.0	4.6	25.9	−13.27	−31.65	8.52
	60	Case 0	93.9	84.4	79.0	59.9	9.6	5.4	19.0	/	/	/
		Case 1	92.6	84.3	80.1	60.3	8.3	4.2	19.7	−12.72	−21.78	3.75
		Case 2	92.7	84.3	80.5	60.3	8.4	3.8	20.2	−12.17	−29.69	6.25
	80	Case 0	106.6	98.7	94.3	79.7	7.9	4.4	14.6	/	/	/
		Case 1	105.1	98.3	94.9	79.9	6.8	3.3	15.0	−12.90	−25.14	2.80
		Case 2	104.9	98.0	95.0	79.4	6.9	3.1	15.5	−12.42	−31.18	6.37
(**b**)												
0	40	Case 0	85.5	73.4	67.0	40.4	12.1	6.4	26.5	/	/	/
		Case 1	84.0	73.8	68.3	40.6	10.2	5.6	27.7	−15.76	−13.13	4.28
		Case 2	84.1	73.9	68.9	40.6	10.2	5.0	28.3	−15.51	−21.26	6.62
	60	Case 0	96.6	86.5	81.2	59.9	10.1	5.3	21.3	/	/	/
		Case 1	95.4	86.7	82.2	60.2	8.7	4.5	22.0	−13.67	−14.70	3.28
		Case 2	95.6	86.9	82.7	60.0	8.8	4.1	22.7	−12.95	−22.11	6.68
	80	Case 0	108.4	100.2	95.9	79.5	8.2	4.3	16.4	/	/	/
		Case 1	107.4	100.2	96.5	79.7	7.2	3.6	16.8	−11.79	−15.58	2.37
		Case 2	107.4	100.1	96.8	79.6	7.3	3.3	17.2	−10.79	−21.96	4.65
90	40	Case 0	85.3	73.3	66.0	40.3	12.0	7.3	25.7	/	/	/
		Case 1	83.5	72.9	67.4	40.7	10.6	5.6	26.7	−11.52	−23.19	3.80
		Case 2	84.1	73.4	68.4	40.8	10.6	5.0	27.6	−11.20	−30.88	7.22
	60	Case 0	96.9	86.8	80.8	60.1	10.1	6.0	20.7	/	/	/
		Case 1	95.3	86.4	81.7	60.0	8.9	4.6	21.7	−11.49	−22.49	4.74
		Case 2	95.5	86.5	82.4	60.3	9.0	4.2	22.1	−10.91	−30.29	6.58
	90	Case 0	108.8	100.5	95.7	79.7	8.3	4.8	16.0	/	/	/
		Case 1	107.5	100.1	96.4	79.7	7.4	3.7	16.7	−11.02	−24.25	4.58
		Case 2	107.5	100.1	96.8	79.7	7.4	3.4	17.0	−11.06	−30.77	6.55

## Data Availability

Data available on request.
